# Circulating miR-18a and miR-532 Levels in Extrahepatic Cholangiocarcinoma

**DOI:** 10.3390/jcm13206177

**Published:** 2024-10-17

**Authors:** Rares Ilie Orzan, Adrian Bogdan Țigu, Vlad-Ionuț Nechita, Madalina Nistor, Renata Agoston, Diana Gonciar, Cristina Pojoga, Andrada Seicean

**Affiliations:** 1 3rd Department of Internal Medicine, “Iuliu Hatieganu” University of Medicine and Pharmacy, Victor Babes Street, No. 8, 400347 Cluj-Napoca, Romania; 2 Regional Institute of Gastroenterology and Hepatology, Croitorilor Street, No. 19–21, 400394 Cluj-Napoca, Romania; 3 Department of Translational Medicine, Institute of Medical Research and Life Sciences—MEDFUTURE, “Iuliu Hatieganu” University of Medicine and Pharmacy, 400337 Cluj-Napoca, Romania; 4 Department of Medical Informatics and Biostatistics, “Iuliu Hațieganu” University of Medicine and Pharmacy, Louis Pasteur Street, No. 6, 400349 Cluj-Napoca, Romania; 5 Faculty of Medicine, “Iuliu Hatieganu” University of Medicine and Pharmacy, Victor Babes Street, No. 8, 400347 Cluj-Napoca, Romania; 6 Pathological Anatomy Discipline, Department of Morphological Sciences, “Iuliu Hatieganu” University of Medicine and Pharmacy, Clinicilor Street, No. 3–5, 400006 Cluj-Napoca, Romania; 7 Department of Clinical Psychology and Psychotherapy, Babeș-Bolyai University, Sindicatelor Street, No. 7, 400029 Cluj-Napoca, Romania

**Keywords:** cholangiocarcinoma, circulating microRNA, biomarkers

## Abstract

**Background**: Cholangiocarcinoma (CCA) is a highly aggressive cancer of the bile ducts with a poor prognosis and limited diagnostic markers. This study aims to investigate the potential of miR-18a and miR-532 as biomarkers for CCA by exploring their correlations with clinical parameters and traditional tumor markers such as CA19.9, CEA, and AFP. **Methods**: This study involved a cohort of patients diagnosed with CCA. Serum levels of miR-18a and miR-532 were measured and analyzed in relation to various clinical parameters, including age, tumor markers, and histological features. **Results**: Serum levels of miR-18a and miR-532 were upregulated in patients with extrahepatic cholangiocarcinoma (eCCA) compared to healthy controls (*p* < 0.05). MiR-18a and miR-532 levels were correlated with each other (*p* = 0.011, Spearman’s rho = 0.482) but showed no significant correlation with age or traditional tumor markers (CA19.9, CEA, AFP). No significant differences in miR-18a and miR-532 levels were observed concerning tumor localization or histological grading. For predicting tumor resectability, miR-532 at a cut-off point of 2.12 showed a sensitivity of 72.73%, specificity of 81.25%, and an AUC of 71.3%, while miR-18a, at a cut-off of 1.83, had a sensitivity of 63.64%, specificity of 75%, and an AUC of 59.7%. ROC curve analysis suggested moderate diagnostic potential for miR-18a and miR-532, with AUC values of 0.64 and 0.689, respectively. **Conclusions**: Although miR-18a and miR-532 showed significant upregulation in eCCA patients compared to healthy controls, they did not demonstrate significant associations with key clinical parameters, limiting their effectiveness as standalone diagnostic biomarkers. Further research involving larger, multi-center cohorts and additional molecular markers is necessary to validate these findings and explore the broader diagnostic potential of miRNAs in CCA.

## 1. Introduction

Cholangiocarcinoma (CCA) encompasses a diverse group of malignancies primarily arising from the epithelial cells lining the bile ducts, although they may also originate from peribiliary glands and hepatocytes [1]. These cancers exhibit significant heterogeneity and are classified into three subtypes based on their primary anatomic locations: intrahepatic CCA (iCCA), perihilar CCA (pCCA), or distal CCA (dCCA) [2]. Extrahepatic cholangiocarcinoma (eCCA) comprises a considerable portion of these tumors, with pCCA being the most prevalent subtype, accounting for 50–60% of all CCAs, followed by dCCA, which represents 20–30% of cases [3]. Each subtype is characterized by several well-established risk factors, as well as distinct molecular and epidemiological features [4]. Despite advancements in diagnostic imaging and therapeutic strategies, CCA remains a challenging disease to diagnose at an early stage and to treat effectively, often presenting resistance to conventional treatments, poor prognosis, and limited survival rates [5,6,7]. 

MicroRNAs (miRNAs) are single-stranded, noncoding RNAs (ncRNAs) that play crucial roles in virtually every cellular process, including apoptosis, proliferation, and differentiation, by directly modulating the expression of tumor suppressor genes and oncogenes [8]. miRNAs are implicated in all steps of biliary carcinogenesis. Upregulated miRNAs can promote cell proliferation and are known as oncogenic miRNAs (e.g., miR-21, miR-191), while others can modulate the oncogenic process and act as tumor suppressors (e.g., miR-34a, miR-122, miR-22). Some miRNAs are utilized as biomarkers for CCA detection (e.g., miR-877, miR-150-5p, miR-21), while others serve as markers of therapy resistance (e.g., miR-199a-3p, miR-106b, miR-130a-3p) [9]. Current diagnostic approaches relying on tissue miRNAs necessitate extraction from human CCA cells, a process that is invasive and not conducive to widespread clinical use. Fortunately, previous reports have suggested that serum miRNAs, which are easily accessible, can serve as biomarkers for diagnosing several cancers with high sensitivity and specificity [10]. Certain miRNAs are stable and easily measurable in serum and plasma, making them promising candidates as non-invasive eCCA biomarkers [11]. Despite these advances, there are few studies evaluating circulating miRNAs specifically as markers of eCCA. Recent integrative genomic analysis of Caucasian eCCA, including pCCA and dCCA, has revealed distinct molecular characterizations of eCCA in Western populations [12]. This is juxtaposed with the epidemiological profiles of CCA and its subtypes, which show significant geographical variation, indicating underlying genomic heterogeneity across different regions [2]. 

In this study, we conducted an analysis of serum miRNA expression in patients diagnosed with extrahepatic cholangiocarcinoma (eCCA). Specifically, our investigation centered on miR-18a and miR-532, which have been implicated in various cancers, such as gastric, colorectal, and hepatocellular carcinomas, but have not been extensively studied in the context of eCCA. Our hypothesis posited that these miRNAs would exhibit differential expression in the serum of eCCA patients, thereby reflecting the unique molecular characteristics of this malignancy. Such findings are crucial for understanding eCCA pathogenesis and hold promise for developing non-invasive diagnostic tools.

## 2. Materials and Methods

### 2.1. Study Design and Ethics

This study was conducted at the Regional Institute of Gastroenterology and Hepatology Cluj-Napoca. The study protocol adhered to the 1975 Declaration of Helsinki guidelines and received approval from the institutional ethics committee before patient inclusion (approval no. 6980 from 23 June 2023). 

### 2.2. Patient Selection

Consecutive patients diagnosed with eCCAs based on tissue acquired from endoscopic ultrasound-guided fine-needle aspiration (EUS-FNA) and confirmed by histological examination were included in the study. For the EUS examinations, we used a linear echoendoscope (Olympus GF-UCT 180 AL5; Olympus, Tokyo, Japan) combined with an ultrasound platform (Hitachi ARIETTA 850). Age- and sex-matched healthy controls were also included for comparison. The inclusion criteria were the following: (1) histologically confirmed diagnosis of eCCA, (2) age 18 years or older, (3) ability to provide written consent, (4) no prior treatment for eCCA, such as chemotherapy, radiotherapy, or surgical intervention, (5) adequate liver and renal function (ALT and AST < 2.5 times the upper limit of normal [ULN], serum creatinine < 1.5 times ULN), (6) performance status of 0–2 on the Eastern Cooperative Oncology Group (ECOG) scale. The exclusion criteria were the following: (1) increased risk of bleeding (international normalized ratio (INR) > 1.5 and/or platelet count < 50,000/mm^3^), (2) history of previous neoplasms, (3) patient refusal to participate, (4) active infection or serious comorbidities that could interfere with the study (e.g., uncontrolled diabetes, severe cardiovascular disease, hepatocellular carcinoma, hepatitis, cholecystitis), (5) known autoimmune diseases or ongoing immunosuppressive therapy, (6) any psychiatric or cognitive condition that would impair the ability to provide informed consent or comply with study requirements.

### 2.3. Sample Collection and miRNA Analysis

Blood samples were collected from all participants under standardized conditions. Venous blood was drawn into serum separator tubes (SST) and allowed to clot for 30 min at room temperature. The samples were then centrifuged at 1500× *g* for 10 min to separate the serum. The serum was aliquoted into RNase-free tubes and stored at −80 °C until further analysis to preserve RNA integrity. Total RNA, including miRNAs, was extracted from 200 µL of serum using the Qiagen miRNeasy Serum/Plasma Kit (Qiagen, Hilden, Germany) according to the manufacturer’s instructions. The concentration and purity of the extracted RNA were assessed using a NanoDrop spectrophotometer (Thermo Fisher Scientific, Wilmington, DE, USA).

The extracted RNA was further processed for cDNA synthesis by reverse transcription using the TaqMan MicroRNA Reverse Transcription Kit (Applied Biosystems, Foster City, CA, USA) and the specific stem-loop primers for miR-18a and miR-532, as well as miR-U6 as control, as presented in Table 1. The reverse transcription was performed on 10 ng total RNA, following the protocol: 30 min at 16 °C, 30 min at 42 °C, and 5 min at 85 °C, then 30 µL of nuclease-free molecular grade water was added to each sample (10 µL for each miR). The RT reaction was performed in a final volume of 10 µL, using 1 µL of reverse transcribed sample with TaqMan Master Mix. The RT was performed on StepOnePlus RT PCR (Applied Biosystems, Foster City, CA, USA), and each sample was run in duplicate following the protocol: initial holding stage 2 min at 50 °C and 20 s at 95 °C, then the cycling stage, including 1 s at 95 °C and 20 s at 60 °C repeated for 40 cycles.

### 2.4. Data Analysis

Relative expression levels of miRNAs were normalized to the endogenous control miRNA using the comparative Ct method (ΔΔCt). The fold changes in miRNA expression between eCCA patients and healthy controls were calculated and log-transformed for statistical analysis. Quality control measures included assessing the amplification efficiency of each qRT-PCR assay and ensuring that the standard curves for each miRNA had an R² value of at least 0.99.

### 2.5. Statistical Analysis

Statistical analysis was performed using R 3.4.3 software statistics [13]. Continuous variables were expressed as mean or median values, according to the distribution, and compared using Student's *t*-test or Mann–Whitney U test, as appropriate. The data collected from the evaluations were tested for normal distribution using a Shapiro–Wilk test for small samples. For multiple comparisons, we included both parametric tests (ANOVA) and non-parametric tests (Kruskall–Wallis) as appropriate. The post hoc analysis was performed with a Games–Howell and a Tukey’s test according to variances. For markers of interest, receiver-operating characteristic (ROC) curve analyses were conducted to determine the cut-off value, significance level, and key reliability indicators of the markers: sensitivity (Se), specificity (Sp), positive predictive value (PPV), and negative predictive value (NPV). The cut-off values were chosen considering the maximum Youden index. The correlations with classical biomarkers were evaluated with Spearman and Kendall rank correlation coefficient in the correlation matrix. Results were considered statistically significant at *p* < 0.05.

## 3. Results

A total of 39 participants were included in this study, consisting of 27 patients diagnosed with eCCA, and 13 age- and sex-matched healthy controls. The demographic and clinical characteristics of the patients included are summarized in Table 2. The main tumor features were determined using EUS or computed tomography (CT) as part of the tumor staging performed at the time of blood sample collection.

### 3.1. miRNA Expression in eCCA Patients and Controls

**Figure 1:**
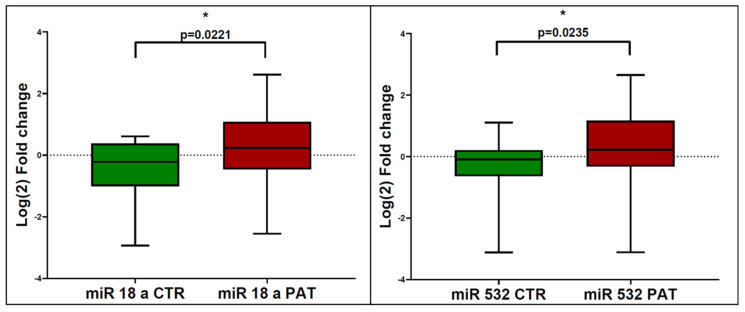
Comparison of serum levels of miR-18a and miR-532 between eCCA patients (PAT) and controls (CTR). The relative expression of circulating miR-18a and miR-532 in serum is expressed as Log(2) of the fold change calculated as 2^−ΔΔCT^ values. The median value for miR-18a in controls is −0.2170 and 0.2404 for patients, while the median value for miR-532 in controls is −0.09518 and 0.2188 for patients. (* *p* < 0.05).

**Figure 2:**
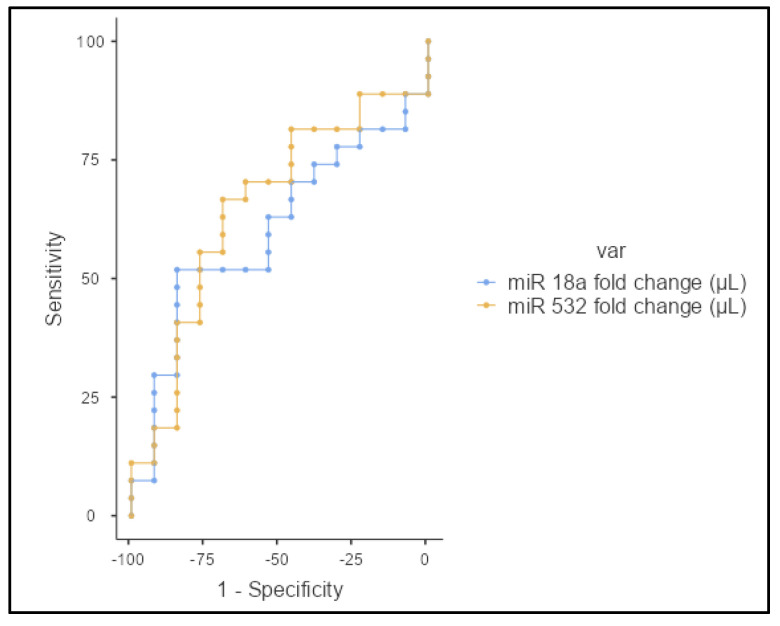
ROC curves for miR-18a (blue) and miR-532 (yellow) in distinguishing between patients with eCCA and healthy controls.

The serum levels of miR-18a were significantly upregulated in patients with eCCA compared to healthy controls. The mean log2 fold change in miR-18a expression was 2.0 in the eCCA group, compared to the control group (*p* = 0.0221). This significant upregulation indicates that miR-18a has a potential role in the molecular pathology of eCCA and has potential as a diagnostic biomarker. Similarly, serum levels of miR-532 were significantly higher in eCCA patients compared to controls. The mean log2 fold change in miR-532 expression was 1.8 in the eCCA group (*p* = 0.0235), suggesting that miR-532 could also serve as a diagnostic biomarker for eCCa (Figure 1). For a miR-18a cutoff of 1.54, the sensitivity was 51.8%, specificity was 84.6%, positive predictive value (PPV) was 87.5%, and negative predictive value (NPV) was 45.8%, with an area under the curve (AUC) of 0.36. For a miR-532 cutoff of 1.1, the sensitivity was 66.6%, specificity was 69.2%, PPV was 80.9%, and NPV was 47.3%, with an AUC of 0.35 (Figure 2).

### 3.2. Correlation and Statistical Analysis

**Figure 3:**
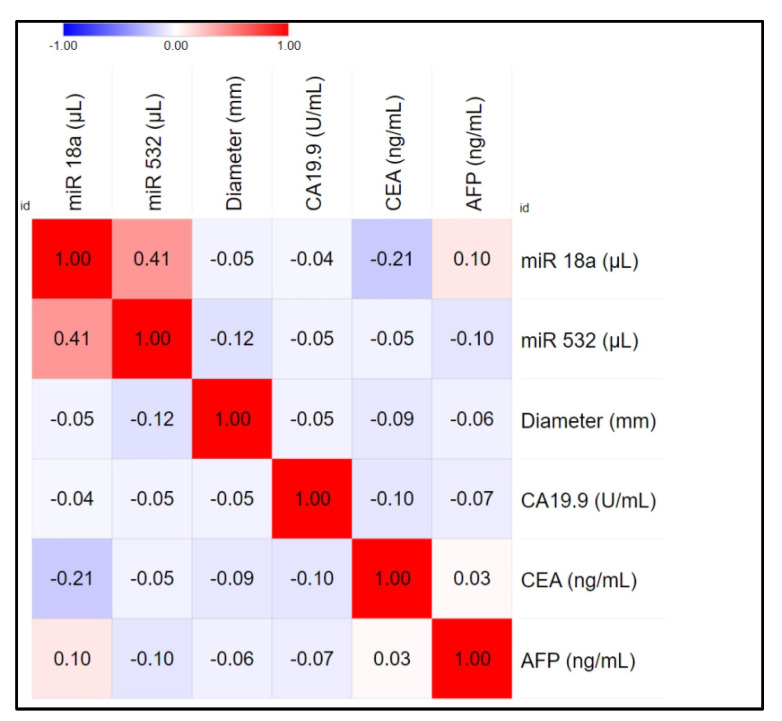
Heat map representing the correlations between miRNA and conventional tumor markers.

The mean serum levels of miR-18a and miR-532 in eCCA patients were 1.54 (SD = 27.2) and 1.67 (SD = 12.5), respectively. The standard deviation (SD) reflects the variability in miRNA levels among the patients. No significant correlations were observed between miRNA levels and patient age (miR-18a: Spearman’s rho = 0.098, *p* = 0.62; miR-532: Spearman’s rho = −0.165, *p* = 0.41). A moderate positive correlation was found between miR-18a and miR-532 levels. Further analysis showed no significant correlations between miRNA levels and tumor markers (CA19.9, CEA, AFP). The correlation matrix with conventional tumor markers is presented in Figure 3. No significant differences were found between the AUCs of AFP, CA19-9, CEA, miR-18a, and miR-532. The AUC values for each marker were analyzed, and while miR-532 showed a slightly higher AUC compared to the others, these differences were not statistically significant.

No significant differences were found in miRNA levels between distal and proximal eCCA using the Mann–Whitney test (*p* = 0.386 for miR-18a and *p* = 0.669 for miR-532). Similarly, there were no significant differences in CA19.9, CEA, and AFP levels with respect to eCCA location (*p* = 0.505, *p* = 0.860, and *p* = 0.615, respectively). Using the Kruskal–Wallis test, miR-18a showed no significant difference between the different histological subgrades (*p* = 0.355; medians: G1 = 1.83, G2 = 1.54, G3 = 0.85). Post hoc analysis indicated *p*-values of 0.99 for G1 vs. G2, 0.79 for G2 vs. G3, and 0.80 for G1 vs. G3. Similarly, miR-532 showed no significant difference between the different histological subgrades (*p* = 0.349; medians: G1 = 2.16, G2 = 1.76, G3 = 1.10). The post hoc test results were *p* = 0.93 for G1 vs. G2, *p* = 0.70 for G2 vs. G3, and *p* = 0.87 for G1 vs. G3. 

### 3.3. Vascular Invasion

**Figure 4:**
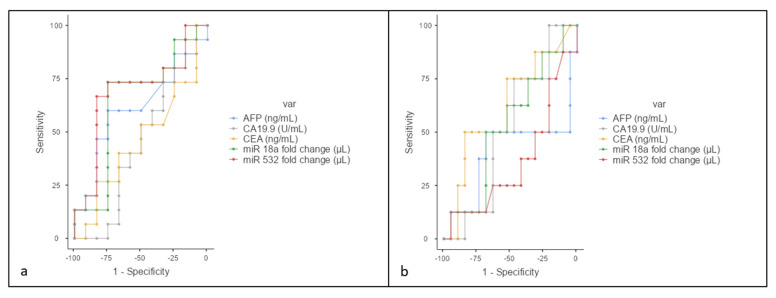
(**a**) ROC curves for AFP (blue), CA19.9 (gray), CEA (yellow), miR-18a (green), and miR-532 (red) in distinguishing between patients with and without vascular invasion. miR-532 demonstrates the highest area under the curve (AUC), indicating a slightly higher differentiation capacity compared to the other markers. (**b**) ROC curve analysis for AFP, CA19.9, CEA, miR-18a, and miR-532 showed varying degrees of sensitivity and specificity in differentiating between different N stages of cholangiocarcinoma.

No significant differences were observed in miR-18a, miR-532, CA19.9, CEA, and AFP levels regarding vascular invasion (miR-18a: *p* = 0.21; miR-532: *p* = 0.10; CA19.9: *p* = 0.64; CEA: *p* = 0.75; AFP: *p* = 0.34). To predict vascular invasion, for a miR-18 cutoff of 1.54, the sensitivity was 73.3%, specificity was 75%, positive predictive value (PPV) was 78.5%, and negative predictive value (NPV) was 69.23%, with an area under the curve (AUC) of 0.64. For a miR-532 cutoff of 1.88, the sensitivity was 66.6%, specificity was 83.3%, PPV was 83.3%, and NPV was 66.6%, with an AUC of 0.689 (Figure 4a). 

### 3.4. N Stage Analysis

No significant differences were found between N0, N1, and N2 stages for miR-18a (*p* = 0.83), miR-532 (*p* = 0.89), CEA (*p* = 0.27), AFP (*p* = 0.64), and CA19.9 (*p* = 0.71). For miR-18a, a cutoff of 2.18 resulted in a sensitivity of 50%, specificity of 68.4%, PPV of 40%, and NPV of 76.4%, with an AUC of 0.53. For miR-532, a cutoff of 27.2 yielded a sensitivity of 12.5%, specificity of 94.7%, PPV of 50%, and NPV of 72%, with an AUC of 0.36 (Figure 4b).

### 3.5. Resectability Evaluation

**Figure 5:**
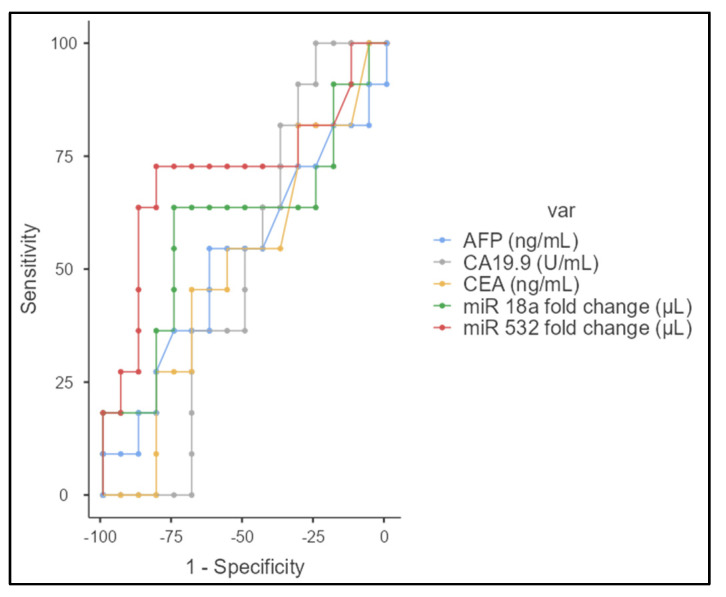
ROC curves for AFP (blue), CA19.9 (gray), CEA (yellow), miR-18a (green), and miR-532 (red) in evaluating resectability.

Regarding the capacity to evaluate tumor resectability, a good prediction model was observed for miR 532 at a cut-off point of 2.12 (Se = 72.73%; Sp = 81.25%; AUC = 71.3%), respectively, for miR 18a at a cut-off 1.83 (Se = 63.64%; Sp = 75%%; AUC = 59.7%) in comparison with classical tumor markers (Figure 5).

## 4. Discussion

In this study, we observed a significant upregulation of miR-18a and miR-532 in the serum of patients with eCCA compared to healthy controls. These findings suggest that these miRNAs might serve as potential biomarkers for the early detection and diagnosis of eCCA.

MiR-18a, part of the miR-17-92 cluster, has been extensively studied in various cancers due to its versatile role in gene regulation [14]. MiR-18a can act as both a tumor suppressor and an oncogene, depending on the cancer type and cellular context [15]. In cancers like breast, colorectal, pancreatic, and hepatocellular carcinoma, miR-18a often exhibits tumor-suppressive properties by targeting critical oncogenes [16,17,18,19]. However, in other malignancies such as gastric and prostate cancers, it promotes oncogenesis by downregulating tumor-suppressor genes [20,21]. 

In intrahepatic cholangiocarcinoma (ICC), the lncRNA MT1JP, known for its tumor-suppressing role, is downregulated and functions by binding to miR-18a-5p, facilitating the expression of fructose-1,6-bisphosphatase 1. Overexpression of MT1JP inhibits cell proliferation, migration, and invasion while inducing apoptosis. This indicates that MT1JP exerts its tumor-suppressing effects in ICC by regulating the miR-18a-5p/FBP1 axis [22]. 

In addition to MT1JP, another lncRNA, CASC2, has been implicated in CCA. CASC2 was found to be expressed at significantly lower levels in CCA tissues and cell lines. Overexpression of CASC2 inhibited cell proliferation, invasion, and migration in CCA cells, while knockdown of CASC2 had the opposite effect. CASC2 functions as a sponge for miR-18a, promoting the expression of SOCS5, a target of miR-18a, thereby inhibiting the epithelial-to-mesenchymal transition progression [23]. The lack of statistical significance in some of our findings could be attributed to the sample size and the inherent biological variability in cholangiocarcinoma. However, the integration of our data with existing literature on the CASC2/miR-18a/SOCS5 axis provides a deeper understanding of the molecular mechanisms underpinning cholangiocarcinoma and highlights potential avenues for therapeutic intervention.

The upregulation of miR-18a in eCCA patients may reflect its role in promoting cell proliferation, inhibiting apoptosis, and enhancing angiogenesis and metastasis. For instance, miR-18a targets and modulates key genes involved in these processes, such as cyclin D1, SMAD2, and HIF-1α, which are critical for cell cycle progression, transforming growth factor-beta (TGF-β) signaling, and hypoxic response, respectively [14,15,24]. This multifaceted role of miR-18a makes it a promising candidate for further investigation as a diagnostic and therapeutic target in eCCA.

Although less studied compared to miR-18a, miR-532 has shown involvement in several cancers, including gastric, colorectal, and hepatocellular carcinomas [25,26,27]. The specific mechanisms by which miR-532 contributes to cancer progression are still being elucidated, but it is known to participate in pathways that regulate cell proliferation, apoptosis, and metastasis. The significant upregulation of miR-532 in our eCCA cohort suggests a potential oncogenic role in this specific type of cholangiocarcinoma.

The upregulation of miR-18a and miR-532 in cholangiocarcinoma was a key finding of this study. Despite their increased expression, no significant correlations were observed between these miRNAs and traditional tumor markers such as CA19.9, CEA, and AFP, nor with histological grading. This suggests that while elevated serum levels of these miRNAs may be associated with eCCA, miR-18a and miR-532 may operate through mechanisms independent of established tumor markers and histopathological features, pointing to a novel regulatory role in cholangiocarcinoma progression. Furthermore, our statistical analysis highlighted the potential of miR-18a and miR-532 in predicting tumor resectability. Specifically, miR-532 demonstrated higher specificity and sensitivity compared to miR-18a. Tumor resectability is traditionally associated with clinical factors such as vascular invasion and lymph node metastasis. However, our study suggests that miR-18a and miR-532 may provide additional predictive value for resectability by capturing early molecular changes that are not immediately detectable through conventional clinical markers. Other miRNAs are involved in regulating key pathways related to tumor proliferation, migration, and invasion, which may signal a more aggressive tumor phenotype before overt clinical signs like vascular invasion or metastasis appear [9]. This could explain why miR-18a and miR-532 demonstrated better performance in predicting resectability despite the absence of significant differences in traditional clinical factors. The AUC values suggest that, while promising, these miRNAs may not be adequate as standalone diagnostic tools. Their utility may be enhanced when combined with other molecular markers or imaging modalities, providing a more comprehensive approach to early detection and surgical decision-making. The observed low AUC values for both miR-18a and miR-532 in our study can be attributed to several factors. Firstly, biological variability plays a significant role; the expression levels of microRNAs can be influenced by individual patient characteristics, disease heterogeneity, and environmental factors, all of which may contribute to the reduced diagnostic performance of these biomarkers. Additionally, the relatively small sample size may limit the statistical power of our analysis, making it difficult to detect significant differences or associations. Cholangiocarcinoma itself is a complex and heterogeneous disease, and while miR-18a and miR-532 may be involved in its pathogenesis, their levels may not accurately reflect the tumor’s biological behavior or progression. Furthermore, the non-specific nature of microRNAs, which can regulate multiple target genes across various biological pathways, may hinder their effectiveness as specific diagnostic markers. Lastly, while these miRNAs show potential, their standalone diagnostic utility appears limited when compared to traditional tumor markers. Acknowledging these factors will enhance the understanding of the limitations and implications of our findings in the context of cholangiocarcinoma diagnosis.

Recent studies suggest that panels of circulating miRNAs may offer superior diagnostic value compared to traditional biomarkers in cancer detection [28]. For instance, Li et al. demonstrated that panels of circulating miRNAs provided greater diagnostic accuracy for hepatocellular carcinoma than conventional biomarkers such as AFP [29]. Similarly, Chen et al. highlighted the potential of circulating miRNAs as novel, highly sensitive biomarkers for cancer diagnosis, suggesting that these miRNA panels could surpass traditional markers in early detection and prognostication [30]. These findings underscore the potential of circulating miRNA panels as a more effective and non-invasive diagnostic tool, particularly in malignancies like cholangiocarcinoma, where early and accurate diagnosis remains challenging.

Several limitations to our study must be acknowledged. Firstly, the sample size is relatively small, which may restrict the generalizability of our findings. Larger cohorts are necessary to validate the observed correlations and trends comprehensively. Additionally, this study was conducted at a single medical center, potentially introducing selection bias. Multi-center studies are warranted to confirm the findings across different populations and healthcare settings. Our results have not been validated in independent cohorts or using different methodologies, such as alternative platforms for miRNA quantification. A limitation of our study is the lack of absolute quantification for miR-18a and miR-532 levels between cholangiocarcinoma (eCCA) patients and controls. We used the 2^−ΔΔCT^ method for relative expression, which, while indicating upregulation, provides only comparative data. The modest differences in median expression values may be insufficient without precise quantification. To better assess the diagnostic value of these miRNAs, a method involving a calibration curve with known miRNA concentrations would allow for accurate, absolute measurements. Future studies should incorporate such quantification to improve the reliability of miRNA biomarkers for cholangiocarcinoma diagnosis. This validation is essential to ensure the robustness and reproducibility of our findings. Moreover, while our study focuses on the correlation between miR-18a, miR-532, and various clinical parameters, it lacks functional assays to explore the biological roles and mechanisms of these miRNAs in cholangiocarcinoma. Investigating whether these elevated miRNAs are produced by eCCA cells or are associated with immune cells involved in the inflammatory response could provide valuable insights. Additionally, exploring the downstream target genes and signaling pathways regulated by miR-18a and miR-532 could offer a clearer understanding of their role in tumor biology and may uncover novel therapeutic or diagnostic targets, thereby increasing the impact of these findings. MiR-18a, along with other miRNAs, may not serve as consistently reliable biomarkers and can only be utilized to a limited extent in clinical practice. McDermott et al. highlighted the challenges in using miRNAs as diagnostic tools, citing issues such as instability and variability, particularly in blood-based studies [31]. This study’s scope is limited to a specific set of miRNAs and traditional tumor markers (CA19.9, CEA, AFP). We did not investigate other miRNAs and molecular markers that might be relevant to cholangiocarcinoma. Additionally, our study relies on specific techniques and platforms for miRNA quantification, which could introduce technical variability. Standardization of protocols and cross-platform comparisons are necessary to ensure consistency in miRNA measurement. Addressing these limitations in future research will be critical for advancing our understanding of miRNA roles in cholangiocarcinoma and improving diagnostic and therapeutic strategies.

## 5. Conclusions

The differential expression of miR-18a and miR-532 in eCCA patients underscores their potential as non-invasive biomarkers for this malignancy. Current diagnostic methods for eCCA, including imaging and histopathological examination, are often insufficient for early detection, leading to delayed treatment and poor prognosis. Using serum miRNAs as biomarkers could enhance early diagnostic capabilities, allowing for timely and more effective therapeutic interventions. Furthermore, understanding the molecular pathways regulated by miR-18a and miR-532 could provide insights into the pathogenesis of eCCA, potentially revealing novel therapeutic targets. Our study highlights the need for further research to validate these findings in larger, independent cohorts and to elucidate the precise biological mechanisms underlying the upregulation of miR-18a and miR-532 in eCCA. Additionally, exploring the interactions between these miRNAs and their target genes could pave the way for new strategies in the management of eCCA.

## Figures and Tables

**Table 1:** The nucleotide sequences for all primers that were used for miRNA expression analysis.

MIR	Mature miRNA Sequence
HSA-miR-18a	ACUGCCCUAAGUGCUCCUUCU
HSA-miR-532	CCUCCCACACCCAAGGCUUGCA
RNU6B	CGCAAGGATGACACGCAAATTCGTGAAGCGTTCCATATTTTT

**Table 2:** Main patient characteristics and tumor features.

Characteristic	eCCA Patients (n = 27)
Age (years), mean ± SD	62.14 (±10.97)
Sex (Male/Female)	14:13
Final diagnosis	
dCCA	10 (37.0%)
pCCA	17 (62.9%)
Histological grading	
G1 (low grade)	9 (33.3%)
G2 (intermediate grade)	13 (48.1%)
G3 (high grade)	5 (18.5%)
Vascular invasion	
None	15 (55.5%)
Present	12 (44.4%)
Visceral invasion	10 (37.0%)
Metastases	4 (14.8%)

eCCA: extrahepatic cholangiocarcinoma; SD: standard deviation; dCCA: distal cholangiocarcinoma; pCCA: proximal cholangiocarcinoma.

## Data Availability

The data presented in this study are available on request from the corresponding author due to privacy and legal reasons.
